# Prospective clinical trial of Intravitreal aflibercept treatment for PolypoIdal choroidal vasculopathy with hemorrhage or exudation (EPIC study): 6 month results

**DOI:** 10.1186/s12886-016-0305-2

**Published:** 2016-07-27

**Authors:** Gregg T. Kokame, James C. Lai, Raymond Wee, Ryan Yanagihara, Jessica G. Shantha, Julia Ayabe, Kelsi Hirai

**Affiliations:** 1 Division of Ophthalmology, Department of Surgery, University of Hawaii School of Medicine, 651 IIalo St, Honolulu, HI 96813 USA; 2 The Retina Center at Pali Momi, 98-1079 Moanalua Road, Suite 470, Aiea, Hawaii 96701 USA; 3 Retina Consultants of Hawaii, 1380 Lusitana St #506, Honolulu, HI 96813 USA; 4 Hawaii Macula and Retina Institute, 98-1079 Moanalua Road, Suite 470, Aiea, Hawaii 96701 USA; 5 John A. Burns School of Medicine, University of Hawaii School of Medicine, 651 Ilalo St, Honolulu, HI 96813 USA

**Keywords:** Choroidal neovascularization, Polypoidal choroidal vasculopathy, Aflibercept, Exudative macular degeneration, Retinal pigment epithelial detachment

## Abstract

**Background:**

Polypoidal choroidal vasculopathy is a variant of choroidal neovascularization and neovascular age related macular degeneration presenting with hemorrhagic and exudative changes within the macula and/or peripapillary region leading to vision loss. In contrast to neovascular age related macular degeneration, polypoidal choroidal vasculopathy has differing clinical manifestations and treatment strategies. Historically, polypoidal choroidal vasculopathy complexes are less responsive to anti-vascular endothelial growth factor therapy with no prospective clinical trials evaluating aflibercept in management of polypoidal choroidal vasculopathy. Herein we prospectively evaluate the efficacy and safety of intravitreal aflibercept in polypoidal choroidal vasculopathy.

**Methods:**

A prospective, open-label, investigator-sponsored trial of intravitreal aflibercept for polypoidal choroidal vasculopathy in 21 eyes was conducted. Injections were administered monthly for 3 initial treatments, then every other month with monthly evaluations. The primary outcome measures were the mean change in best corrected visual acuity and adverse events. Secondary outcome measures included stabilization of vision, presence of subretinal hemorrhage, serous detachment, retinal pigment epithelial detachment, and regression of polypoidal complexes on indocyanine green angiography.

**Results:**

At 6 months, the median visual acuity was 20/40 (range 20/25–20/200) with a mean Early Treatment Diabetic Retinopathy Study vision of 68.4 letters. There was a gain of 2.76 Early Treatment Diabetic Retinopathy Study letters at 6 months (*p* = 0.15). No patient developed severe vision loss (≤15 letters) and vision was stable or improved in 19/21 eyes (91 %). Subretinal fluid resolved in 13/18 eyes (72 %), and subretinal hemorrhage resolved in 6/8 eyes (75 %) respectively. The polyps regressed in 14/21 eyes (67 %) and the branching vascular network decreased in 1 eye and was stable in all other eyes. The retinal pigment epithelial detachment improved in 13/15 eyes (87 %). Bimonthly treatment occurred in 15/21 patients (71 %). There were no adverse events.

**Conclusions:**

Intravitreal aflibercept results in stabilization of vision, resolution of exudative and hemorrhagic complications with regression of polyps in polypoidal choroidal vasculopathy. Eyes with polypoidal choroidal vasculopathy previously treated with ranibizumab and bevacizumab can show marked improvement in the retinal pigment epithelial detachments and persistent polyps with aflibercept therapy.

**Trial registration:**

Clinical trials.gov NCT01871376, June 4^th^ 2013

**Electronic supplementary material:**

The online version of this article (doi:10.1186/s12886-016-0305-2) contains supplementary material, which is available to authorized users.

## Background

Polypoidal choroidal vasculopathy (PCV) is increasingly recognized as a cause of exudative and hemorrhagic complications in the macula [1–3]. Patients with PCV present with subretinal hemorrhage, subretinal fluid, and retinal pigment epithelial detachment (RPED). These findings are associated with a subretinal branching vascular network (BVN) with characteristic “polyplike” structures at the terminal ends of the vessels [1–5]. Since many of the fundus and optical coherence tomography (OCT) findings are similar to typical exudative age related macular degeneration (AMD), indocyanine green (ICG) angiography is essential to diagnosing PCV [1, 5]. Polypoidal choroidal vasculopathy is a variant of type I subretinal neovascularization (Gass classification) [6], in which the neovascular complex grows beneath the retinal pigment epithelium (RPE) and above Bruch’s membrane [7–9].

The treatment of the hemorrhagic and exudative complications of PCV is not well defined, but therapeutic approaches include photodynamic therapy (PDT), anti-vascular endothelial growth factor (VEGF) therapy, combined PDT and anti-VEGF therapy, direct thermal laser to polyps, and surgical management of significant subretinal hemorrhage or hemorrhagic retinal detachment [10]. Photodynamic therapy has been the mainstay of treatment in Asia for years [11–14], and was supported by an expert panel on PDT management in Asia. This treatment is usually guided by the size of the complex on ICG angiography. [11]. Polypoidal choroidal vasculopathy is much more common in Asian populations and is being increasingly recognized in White populations with more frequent use of ICG angiography [3, 15–20].

Anti-VEGF therapy has recently been shown to be effective in decreasing the exudation, macular edema, and hemorrhage associated with PCV [12, 21–25]. Although retrospective studies are numerous, there are very limited prospective studies available for evaluation of the different anti-VEGF agents. For ranibizumab treatment in PCV, there are 6 month prospective results available in the Efficacy and safety of verteporfin photodynamic therapy in combination with ranibizumab or alone versus ranibizumab monotherapy in patients with symptomatic macular polypoidal choroidal vasculopathy trial (EVEREST trial ranibizumab 0.5 mg) [12], in the investigator sponsored trial for Polypoidal Choroidal Vasculopathy with Intravitreal Ranibizumab (PEARL trial, ranibizumab 0.5 mg) [21, 23], and in the investigator sponsored trial of Polypoidal Choroidal Vasculopathy Evaluation Assessing High-Dose Ranibizumab Prospectively (PEARL2 trial, 2.0 mg ranibizumab) [8]. For aflibercept, only one recent prospective study of aflibercept 2.0 mg exists in treatment naïve eyes with exudative or hemorrhagic PCV [22]. The Prospective Clinical Trial of Intravitreal Aflibercept Treatment for PolypoIdal Choroidal Vasculopathy with Hemorrhage or Exudation trial (EPIC trial) results reported herein provides 6 month results on eyes with PCV that were both treatment naïve, as well as eyes previously treated with anti-angiogenic therapy, including treatment with high dose ranibizumab therapy (ranibizumab 1.0 mg or 2.0 mg) for 24 months in the PEARL2 trial.

## Methods

The EPIC trial (Intravitreal Aflibercept Injections (**E**ylea®) for **P**olypo**i**dal **C**horoidal Vasculopathy with Hemorrhage or Exudation) is an ongoing, prospective, open-label clinical trial of intravitreal aflibercept (2.0 mg/0.05 mL) in patients with active hemorrhage, exudation, or recent decrease in vision (defined as a loss of five Early Treatment Diabetic Retinopathy Study letters (ETDRS) or one Snellen line of vision in the past 6 months) associated with PCV. Major exclusion criteria include: (1) history of previous vitrectomy; (2) previous cataract surgery within 2 months prior to the baseline visit; (3) presence of any condition that would jeopardize the patient’s participation in this study; (4) no prior anti-VEGF (pegaptanib, bevacizumab, ranibizumab, aflibercept) in study eye within 30 days of enrollment; (5) known allergy to the study drug; (6) poorly controlled hypertension; (7) major surgery within 28 days prior to study. The EPIC study was approved by the Western Institutional Review Board (IRB, Puyallup, Washington, Study Number 1138211) and listed on clinical trials.gov (NCT01871376). The study was carried out at The Retina Center at Pali Momi. It adhered to the tenets of the guidelines of the Declaration of Helsinki, and each patient completed a written informed consent. The EPIC trial also followed the TREND guidelines/methodology. This study was supported in part as an investigator-sponsored trial by a research grant from Regeneron, Inc (Tarrytown, New York). The investigators maintained complete control of the data and its interpretation.

**Fig. 1:**
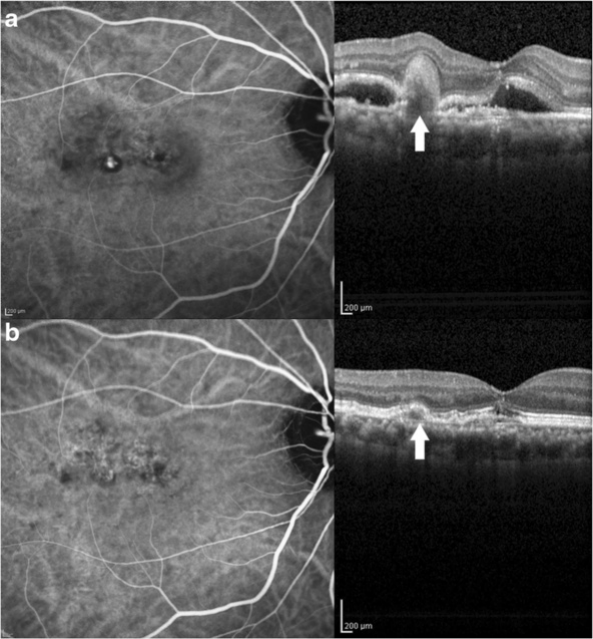
Marked reduction of treatment-naïve polyp after 6 months of intravitreal aflibercept. **a**. EPIC study baseline ICG angiogram (left) with correlated OCT study (right). Note the hyperfluorescent polyp with hypofluorescent ring on ICG angiogram. On the OCT, an inverted U-shaped polyp (arrow) is seen with surrounding serous detachment. **b**. ICG angiogram (left) following 6 months of intravitreal aflibercept with corresponding OCT (right) reveals marked reduction in the polyp (arrow)

The clinical diagnosis of PCV was based on funduscopic identification of serous retinal detachment, macular edema, subretinal hemorrhage, or RPED. The definitive diagnosis was confirmed by ICG angiography, in which PCV complexes were identified as polyps with or without a BVN (Fig. 1). All patients received multiple, open-label intravitreal injections of 2.0 mg aflibercept (Regeneron, Tarrytown, New York, United States) administered per protocol for 6 months. The first 3 months required monthly mandatory dosing (30 days + 7 days) (baseline, month 1, month 2). Subsequent injections were administered every other month (60 days ± 7 days). If needed, patients were allowed monthly treatment (30 days ± 7 days), if there was recurrence or persistence of subretinal fluid, subretinal hemorrhage or macular edema with or without vision loss. Recurrence of subretinal fluid or hemorrhage after previous resolution of subretinal fluid or hemorrhage required three consecutive monthly mandatory injections before a possible monthly examination without treatment could be considered.

At baseline all patients had a complete ophthalmic examination with best corrected visual acuity determined with ETDRS charts and refraction at 4 m (BCVA), slit-lamp examination, funduscopic biomicroscopic examination, fundus photography, fundus autofluorescence testing, fluorescein angiography (FA), and ICG angiography using the scanning laser ophthalmoscope (Heidelberg Spectralis HRA + OCT, Heidelberg Engineering USA, Carlsbad, California) and optical coherence tomography (OCT) with point-to-point localization of the OCT with the FA and ICG angiography landmarks. Ophthalmic examination, visual acuity, fundus autofluorescence (FAF), and OCT were performed monthly. Early Treatment Diabetic Retinopathy Study vision at 4 m, FA, and ICG angiography were performed at baseline, and at 1,3, and 6 months. Eyes will be followed in the study for 24 months.

The primary outcome measures are the mean change in BCVA and the incidence and severity of ocular and systemic adverse events. Secondary outcome measures included stabilization of vision (loss of <15 ETDRS letters), changes in subretinal fluid, presence of subretinal hemorrhage, decrease in RPED, change in central foveal thickness (CFT), presence of macular edema, leakage on fluorescein angiography, and regression of polypoidal complexes on ICG angiography. The PCV complex was composed of polyp lesions and a BVN in all patients. Both components were evaluated separately. Decrease in polyp lesion or BVN was any decrease in size or number of polyps as compared to baseline imaging. Stable findings of the polyp or BVN indicates no change in size or number of polyps in response to therapy. Regression and/or resolution of the polyp indicates that it has resolved on ICG. Macular edema was defined as an increase in macular thickness including subretinal fluid and intraretinal edema as visualized on OCT. Paired t-tests were performed to compare baseline and 6 month ETDRS best corrected visual acuity and change in OCT CFT. Statistical significance was based on p values <0.05 and the statistical analysis was performed using SAS 9.2 software (Cary, North Carolina).

## Results

**Fig. 2:**
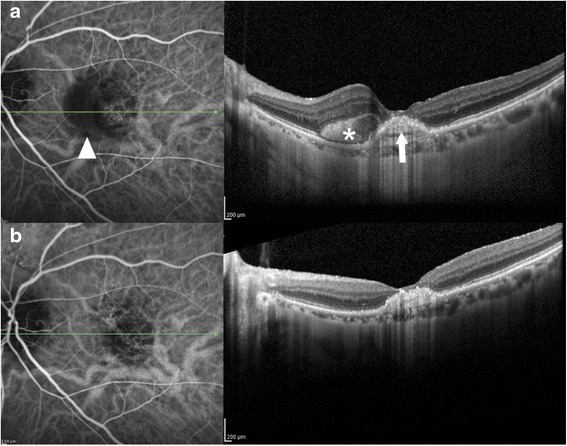
ICG angiogram with correlated OCT study in a previously treated eye in the PEARL 2 Study (12 2.0 mg ranibizumab + 12 1.0 mg ranibizumab) with additional bevacizumab intravitreal injections prior to entry into the EPIC study. **a**. Baseline EPIC Study images. ICG angiogram shows hypofluorescence (see arrowhead) in the area of the subretinal hyperreflective material and the central RPED. OCT shows a central RPED (arrow) with nasal subretinal hyperreflective material (asterisk). **b**. EPIC Month 6 imaging. ICG angiogram shows decrease in the area of hypofluorescence. OCT shows resolution of the subretinal hyperreflective material and decrease in the RPED. Visual acuity improved from 20/160 to 20/63 (+30 letters)

Baseline characteristics of patients entered into the EPIC trial can be found in Table 1. Patients were treated with bevacizumab and/or ranibizumab with a range of 1 to 36 injections in the 11 patients previously treated with anti-VEGF therapy. Before entering the EPIC trial, patients did not receive an injection within 30 days of screening and had active disease at entry. The one patient with prior PDT had two PDT treatments over 2 years before entering the EPIC trial and had developed recurrent leakage and serous detachment upon entry. All patients with active leakage or bleeding associated with PCV were offered entry into the EPIC trial.. Baseline findings included subretinal fluid in 18/21 eyes (86), subretinal/sub-RPE hemorrhage in 8/21 eyes (38), and RPED in 15/21 eyes (71 %). On ICG angiography polypoidal complexes were in the macula in 17 eyes (81), in the peripapillary region alone in 1 eye (5), and in both the macular and peripapillary regions in 3 eyes (14 %) at the baseline EPIC study visit. The median BCVA was 20/40 (range: 20/16 – 20/200) at baseline and 20/40 (range: 20/25 – 20/200) at 6 months. The BCVA was 20/32 or better at baseline in 11/21 eyes (52 %). The range of vision in the remaining 10 eyes was from 20/40 to 20/200. The mean ETDRS BCVA was 65.7 letters at baseline, and 68.4 letters at 6 months (Table 2). The average gain in ETDRS letters over 6 months was 2.76 letters (*p* = 0.15). None of the patients lost ≥ 15 letters in ETDRS vision at 6 months. One patient (5 %) gained ≥ 15 letters at 6 months (Fig. 2). Six eyes (29) improved ≥ 5 letters, thirteen eyes (61) remained unchanged (<5 letter change) from baseline, and two eyes (10 %) decreased ≥ 5 letters. The changes in ETDRS letters based on previous treatment can be found in Table 3.

**Table 1:** Baseline Characteristics of the EPIC Study

Characteristic	(*N* = 21)
Mean Age, Years (Range)	77 (59 – 91)
Males, N (%)	10 (48)
Ethnicity, N (%)	
Asian	19 (90)
Caucasian	2 (10)
Laterality, N (%)	
Unilateral	16 (76)
Bilateral	5 (24)
Prior Treatment, N (%)	
Prior Anti-VEGF	11 (52)
Prior ML	1 (4)
Prior PDT	1 (4)
None (Treatment-Naïve)	10 (48)
Median Visual Acuity (Range)	20/40 (20/200 – 20/16)
Mean ETDRS Letters (Range)	65.7 (30 – 89)
Mean Central Foveal Thickness, (μM)	283 (164 – 550)

*VEGF* vascular endothelial growth factor, *ML* macular laser, *PDT* Photodynamic therapy, *ETDRS* Early Treatment Diabetic Retinopathy Study, *μm* micrometers, *n* number

**Table 2:** Comparisons of change in clinical manifestations at baseline and 6 months

Patients	ETDRS letters baseline	ETDRS letters 6 months	CFT baseline (μM)	CFT 6 months (μM)	Polyps At 6 months (ICG)	BVN At 6 months (ICG)
1	71	70	210	199	=	=
2	30	60	176	119	↓	=
3	72	68	230	235	R	=
4	75	73	398	177	=	=
5	48	49	276	218	↓	=
6	75	68	318	188	=	=
7	58	72	278	289	=	=
8	59	68	352	343	=	=
9	74	83	237	227	R	=
10	82	80	550	198	↓	↓
11	80	81	249	183	R	=
12	38	37	208	193	↓	=
13	60	67	317	227	R	=
14	36	38	387	163	↓	=
15	83	80	223	200	=	=
16	73	77	222	221	↑	=
17	74	76	346	220	R	=
18	89	83	196	178	↓	=
19	74	73	290	223	↓	=
20	61	70	315	190	↓	=
21	67	64	164	158	R	=

*CFT* Central foveal thickness, *ETDRS*: Early Treatment Diabetic Retinopathy Study, *BVN*: Branching vascular network, *ICG*: Indocyanine green angiography, ↑ = increased, ↓ = decreased, = =stable, *R* regressed

**Table 3:** Comparison of change in ETDRS from baseline 6 months of treatment in the EPIC trial

Change in ETDRS letters	Treatment naïve (%)	Previously treated (%)	Total
Improved ≥ 5 Letters	3 (30)	3 (27)	6
Unchanged < 5 Letters Change	6 (60)	7 (64)	13
Decreased ≥ 5 Letters	1 (10)	1 (9)	2
Increase ≥ 15 Letters	0	0	0
Decrease ≥ 15 Letters	0	1 (9)	1

*ETDRS* Early Treatment Diabetic Retinopathy Study

**Fig. 3:**
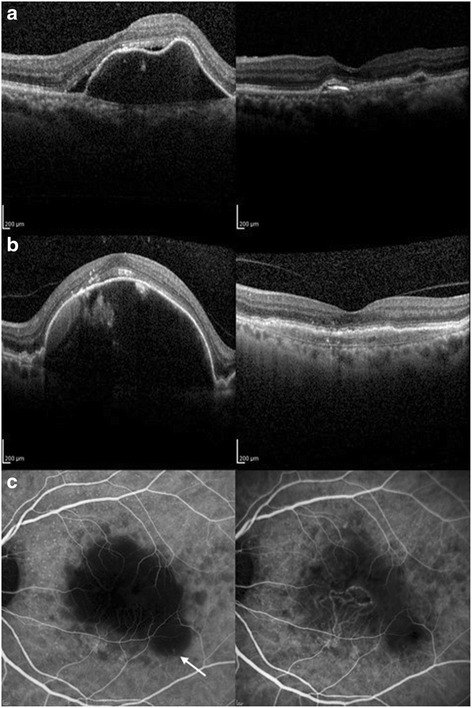
Resolution of a vascularized RPED after aflibercept therapy. **a**. An Asian male with a persistent large vascularized RPED after 24 months of high dose ranibizumab therapy (12 2.0 mg ranibizumab + 12 1.0 mg ranibizumab injections) in PEARL2 study at EPIC baseline (upper left). Resolution of the vascularized RPED which initially resolved at month 3 and stayed resolved at month 6 (upper right) **b**. Treatment naïve Caucasian female with vascularized RPED at EPIC baseline (left) and after aflibercept therapy at month 6 (right). Note the marked improvement in the vascularized RPED. **c**. Corresponding ICG angiogram to patient in B. Increased visibility of BVN after RPED resolution on aflibercept therapy. ICG angiogram shows hypofluorescence in the area of the RPED at baseline with inferior PCV complex (left). Follow-up ICG angiogram shows decreased hypofluorescence due to resolution of the RPED. Note the increased visibility of the hyperfluorescent BVN (right)

At 6 months, subretinal hemorrhage resolved in 6/8 eyes (75), persisted in 1/8 eyes (12.5), and increased in 1/8 eyes (12.5 %). Subretinal fluid completely resolved in 13/18 eyes (72 %), decreased in 3/18 eyes (17 %), and remained stable in 2/18 eyes (11 %). A statistical significant decrease was noted in the comparison of the average CFT at baseline (282 μm) and at 6 months (207 μm) with an average decrease of 75 μm (*p* = 0.0014, Table 2). The RPED resolved completely or decreased in 13/15 eyes (87 %) (Fig. 3), stable in 1/15 eyes (7 %), and increased in 1/15 eyes (7 %). Significant macular edema at baseline due to PCV was present in 17/21 eyes (81 %). In these 17 eyes, macular edema improved in 13/17 eyes (76 %). In 4 cases, the edema remained stable.

At 6 months 15/21 eyes (71 %) received treatment every other month per EPIC protocol. The remaining 6 eyes (29 %) were receiving monthly injections at month 6. This included 3 eyes with 6 monthly injections, and 3 eyes with 5 injections. Two patients had recurrences both occurring at month 4. These recurrences then necessitated restarting monthly therapy for 3 aflibercept treatments. One patient missed a follow-up evaluation at month 3. This patient had persistent subretinal fluid throughout the study and required monthly treatments..Of the 15 eyes on every other month treatment at month 6, only one of the fifteen eyes developed recurrent subretinal hemorrhage at the month 6 evaluation. The other 14 eyes had resolved subretinal fluid and subretinal hemorrhage at month 6 on every other month therapy.

**Fig. 4:**
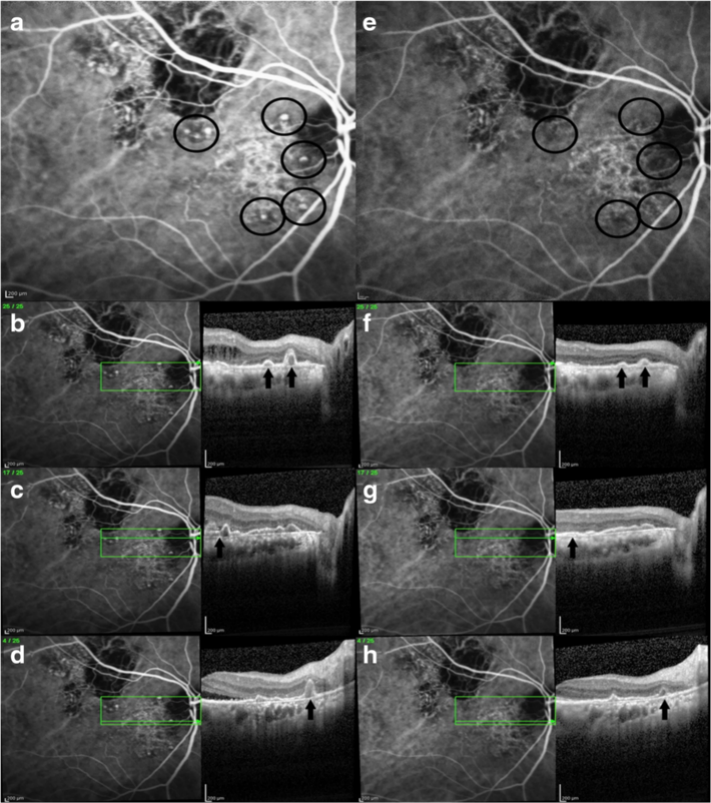
Resolution of polyps on aflibercept in an Asian Male with a large vascular PCV complex persisting after 24 months of high dose ranibizumab therapy (PEARL2, 6 2.0 mg ranibizumab + 18 1.0 mg ranibizumab injections). **a**. EPIC baseline ICG angiogram showing persistent polyps (circles). **b**-**d**. EPIC baseline images with ICG angiogram and corresponding polyps (arrows) on OCT. **e**. Epic month 6 ICG angiogram showing marked polyp resolution. **f**-**h**. EPIC month 6 ICG angiogram with corresponding OCT images displaying significant polyp (arrows) reduction on aflibercept therapy

The response of the polypoidal complex to aflibercept therapy utilizing the EPIC protocol was evaluated with ICG angiography (Table 2). Polyps resolved or decreased in 14/21 eyes (67 %) (Figs. 1, 4). Complete polyp regression was noted in 6/21 eyes (29 %). Polyps remained stable in 6/21 (29 %) eyes. In one eye (5 %), there was a localized increase in the inferior peripheral polyps along the PCV complex on aflibercept therapy, the BVN remained stable in size without associated bleeding or leaking at 6 months. The BVN remained the same size in 20 eyes (95) and decreased in one eye (5 %). In the 15 eyes presenting with RPED, the ICG angiogram looks hypofluorescent in the area of the RPED. However, if the RPED flattens, the polypoidal complex may then show increased visibility of the BVN (Fig. 3c). This was noted in 9 of the 15 eyes (60 %) initially presenting with RPED, where 13 eyes showed significant flattening of the RPED (Fig. 3 a, b). In one eye that had received 24 months of high dose ranibizumab in the PEARL2 study, there was a marked reduction in polyps on aflibercept at 6 months (Fig. 4).

On fundus autofluorescence at baseline polyps were seen as hyperauthofluorescent with a surrounding hypoautofluorescent ring in 9 eyes, hyperautofluorescent in 3 eyes, hypoautofluorescent in 6 eyes, and hypoautofluorescent with hyperautofluorescent ring in 2 eyes. The BVN was visualized as hypoautofluorescent in 13 eyes, hyperautofluorescent in 6 eyes and not visualized 2 eyes respectively. At 6 months polyps were not visualized in 10 eyes, hyperautofluorescent in 4 eyes, hypoautofluorescent in 5 eyes, hypoautofluorescent with hyperautofluorescent ring in 2 eyes. The BVN’s at 6 months were hypoautofluorescent in 14 eyes,, hyperautofluorescent in 3 eyes, and not visualized in 4 eyes. The BVN’s in these patients had indistinct borders. At baseline 4 patients had blocked autofluorescence due to SRH which resolved in 3 patients at 6 months. In 4 patients there were areas of diffuse hyper-autofluorescence within the macula.

There were no ocular or systemic adverse events noted in the study at 6 months.

## Discussion

Polypoidal choroidal vasculopathy is a major cause of leaking and bleeding in the macula and may respond differently to therapeutic agents than typical exudative AMD [8, 9]. In the EPIC study, aflibercept was evaluated in an IRB-approved, prospective, clinical trial as an intravitreal therapy for PCV in eyes with confirmation of the PCV diagnosis by ICG angiography. Significant resolution of leaking and bleeding was noted in response to aflibercept. A statistical significant decrease in CFT was observed in these subset of patients. In addition, significant reduction was noted in RPED in 87 % of cases (Fig. 3). In the PEARL study (ranibizumab 0.5 mg) there was a 50 % reduction in RPED [21], and in the PEARL2 study (2.0 mg ranibizumab) there was a 63 % reduction in RPED [8]. In one patient entering the EPIC study there was a persistent large RPED after 24 monthly high dose ranibizumab injections in the PEARL2 study. After entering the EPIC trial with aflibercept, the highly elevated vascularized RPED resolved at 3 months and remained flat at month 6 with maintenance of stable vision (Fig. 3a). A greater resolution of RPED has also been noted after switching from bevacizumab and ranibizumab to aflibercept in multiple studies on therapy for wet AMD [26, 27]. ICG angiography was not performed in these studies, but vascularized RPED is more frequent in eyes with PCV than in eyes with typical exudative AMD.

Previous studies have demonstrated a higher rate of polyp regression in therapies utilizing PDT, or combination PDT and intravitreal injection of antiangiogenic drug. In the EVEREST study at 6 months there was a 77.8 % polyp closure rate with PDT + ranibizumab, a 71.4 % polyp closure rate with PDT alone, and a 28.6 % polyp closure rate with ranibizumab monotherapy [12]. However, there was not a statistically significant difference in vision results. The PEARL trial confirmed a relatively low rate of polyp regression of 33 % at 6 months [21], and 38 % after one year of monthly 0.5 mg ranibizumab therapy [23]. The PEARL, PEARL2 and EPIC studies are different prospective trials with different agents and different patient populations with relatively small sample sizes, so comparison between studies cannot be directly made. However, the ETDRS vision testing, OCT evaluation, and ICG angiography evaluations were consistent across all 3 studies. The anatomic data does show a higher polyp regression rate in the EPIC study of 67 %. In the PEARL study, the polyp regression rate was 33 % at 6 months [21] and in the PEARL2 study the polyp regression rate was 63 % at 6 months [8], but this dose is not commercially available. In one patient previously treated with high dose ranibizumab in the PEARL2 study, there were marked persistent polyps, which markedly regressed after 6 months of treatment with aflibercept in this EPIC study (Fig. 4).

Because half of the eyes entering into the EPIC study had a BCVAof 20/32 or better, a significant improvement in vision (>15 letters) could not be demonstrated in these eyes due to the ceiling effect. There was not a statistically significant improvement in mean BCVA in this patient population with good baseline vision, but the primary endpoint for vision showed that none of the patients developed severe vision loss (>15 letters). The great majority of eyes showed stability of vision with 90 % showing no loss of vision of greater than 5 ETDRS letters after 6 months.

PCV tends to have a higher incidence of anti-VEGF resistance [19], but in the EPIC study 71 % of eyes were able to be treated with every other month therapy at month 6 after the initial 3 monthly aflibercept treatments. Only one of these eyes developed recurrence at month 6, and then required monthly therapy. In one case treated in the PEARL2 study after 24 months of monthly high dose ranibizumab therapy there was marked resolution of persistent polyps on therapy with aflibercept (Fig. 4). This high rate of polyp closure with aflibercept was confirmed in the recent study by Hosokawa and colleagues with a 77.7 % resolution rate of polyps [22]. In another patient that was previously treated with ranibizumab in the PEARL2 Study and intravitreal bevacizumab, a persistent RPED with hyperreflective material was noted prior to entry into the EPIC trial. After treatment with aflibercept the central RPED decreased with resolution of the subretinal hyperreflective material and BCVA improvement from 20/160 to 20/63. This patient had a better anatomical and visual acuity response to aflibercept therapy than the previous anti-VEGF therapies highlighting the use of aflibercept in PCV patients that may not respond completely to other agents (Fig. 2).

Fundus autofluorescence was performed in all patients. At baseline there was more abnormalities as previously described by Koizumi as hyperautofluorescent lesions with a hypoautofluorescent ring, but this was less noticeable after treatment. This technique of imaging produced varied results of the characteristic polyps and BVN’s when compared to ICG. The BVN in all patients had indistinct borders making it difficult to distinguish lesion extent. In select patients areas of diffuse hyper-autofluorescence were noted corresponding to areas of previous leakage with RPE stress [28].

While this study was designed as a prospective, open-label, clinical trial there are still limitations present. One is the small sample size, in which larger prospective clinical trials with treatment of PCV are necessary to further define and validate the appropriate treatment algorithms. This study included patients that have been previously treated with PDT and/or anti-VEGF therapy which might negate responses to the current therapy. Lastly, the initial visual acuity criteria being very good at entry led to the inability to detect a statistical significant change in vision after treatment due to the ceiling effect.

The EPIC results are promising for the treatment of PCV in resolution of the leaking and bleeding complications of PCV, as well as in regards to increased resolution of the PCV complex. In addition, duration of therapy may allow less frequent dosing, as 71 % of cases were treated with every other month therapy by month 6. Possible etiologies for this increased effect in PCV are the higher binding affinity of aflibercept, as well as possible active transport of the aflibercept molecule across the RPE cells. As shown in pre-clinical studies, ranibizumab depends on a gradient diffusion between RPE cells, whereas aflibercept is actively transported to the sub-retinal pigment epithelial space by RPE cells [29].

## Conclusion

Intravitreal aflibercept therapy is an effective treatment in PCV. It can stabilize vision, resolve exudative and hemorrhagic complications in the macula, and promote polyp regression within the PCV complex.. Future studies with longer term results and larger numbers of patients will help to further guide aflibercept’s role in PCV management.

## Abbreviations

AMD, age related macular degeneration; BVN, branching vascular network: CFT, central foveal thickness: EPIC, prospective clinical trial of intravitreal aflibercept treatment for polypoidal choroidal vasculopathy with hemorrhage or exudation: ETDRS, Early Treatment Diabetic Retinopathy Study: EVEREST, efficacy and safety of verteporfin photodynamic therapy in combination with ranibizumab or alone versus ranibizumab monotherapy in patients with symptomatic macular polypoidal choroidal vasculopathy: FA, fluorescein angiography; ICG, indocyanine green angiography; IRB, Institutional Review Board; OCT, optical coherence tomography: PCV, polypoidal choroidal vasculopathy; NVAMD, neovascular age related macular degeneration; PDT, photodynamic therapy; PEARL 2, investigator sponsored trial of Polypoidal Choroidal Vasculopathy Evaluation Assessing High-Dose Ranibizumab Prospectively: PEARL, investigator sponsored trial for polypoidal choroidal vasculopathy with Intravitreal Ranibizumab: RPE, retinal pigment epithelium; RPED, retinal pigment epithelial detachment; VEGF, vascular endothelial growth factor

## Additional file

Additional file 1: Epic 6 Month Results. The data in this supplementary table consists of both descriptive anatomical descriptions and quantitative data that was used to determine the effect aflibercept therapy had on the hemorrhagic and exudative changes within the macula. (XLS 107 kb)
